# Birdsong fails to support object categorization in human infants

**DOI:** 10.1371/journal.pone.0247430

**Published:** 2021-03-11

**Authors:** Kali Woodruff Carr, Danielle R. Perszyk, Sandra R. Waxman

**Affiliations:** 1 Department of Psychology, Northwestern University, Evanston, Illinois, United States of America; 2 Department of Communication Sciences and Disorders, Northwestern University, Evanston, Illinois, United States of America; 3 Institute for Innovations in Developmental Sciences, Northwestern University, Evanston, Illinois, United States of America; 4 Institute for Policy Research, Northwestern University, Evanston, Illinois, United States of America; Texas Christian University, UNITED STATES

## Abstract

Recent evidence reveals a precocious link between language and cognition in human infants: listening to their native language supports infants’ core cognitive processes, including object categorization, and does so in a way that other acoustic signals (e.g., time-reversed speech; sine-wave tone sequences) do not. Moreover, language is not the only signal that confers this cognitive advantage: listening to vocalizations of non-human primates also supports object categorization in 3- and 4-month-olds. Here, we move beyond primate vocalizations to clarify the breadth of acoustic signals that promote infant cognition. We ask whether listening to birdsong, another naturally produced animal vocalization, also supports object categorization in 3- and 4-month-old infants. We report that listening to zebra finch song failed to confer a cognitive advantage. This outcome brings us closer to identifying a boundary condition on the range of non-linguistic acoustic signals that initially support infant cognition.

## Introduction

The power of human language derives in large part from its inextricable link to cognition (for reviews, see [1–4]. Much of the developmental evidence concerning the acquisition of this uniquely human link has been based on the discovery that listening to language boosts infants’ success in core cognitive capacities including abstract rule-learning [5] and object categorization [6].

The focus on object categorization is especially apt because it is a fundamental building block of cognition [7–9]. Categorization influences virtually all aspects of learning and cognition across species and across development: when we construe two objects as members of the same category, we establish their equivalence at a certain level of representation. This representation permits us to identify new category members and to make category-based inferences. This has powerful consequences for subsequent learning. For example, once infants represent distinct individuals as members of a category CAT, they can learn from a single encounter with a single cat that it is best to avoid pulling the tail of other cats. Object categories also support memory and reasoning, guiding our predictions about properties of objects—even objects we may have never encountered (e.g., a hairless cat) and properties of objects we may never observe directly (e.g., its DNA) [9–11].

**Fig 1:**
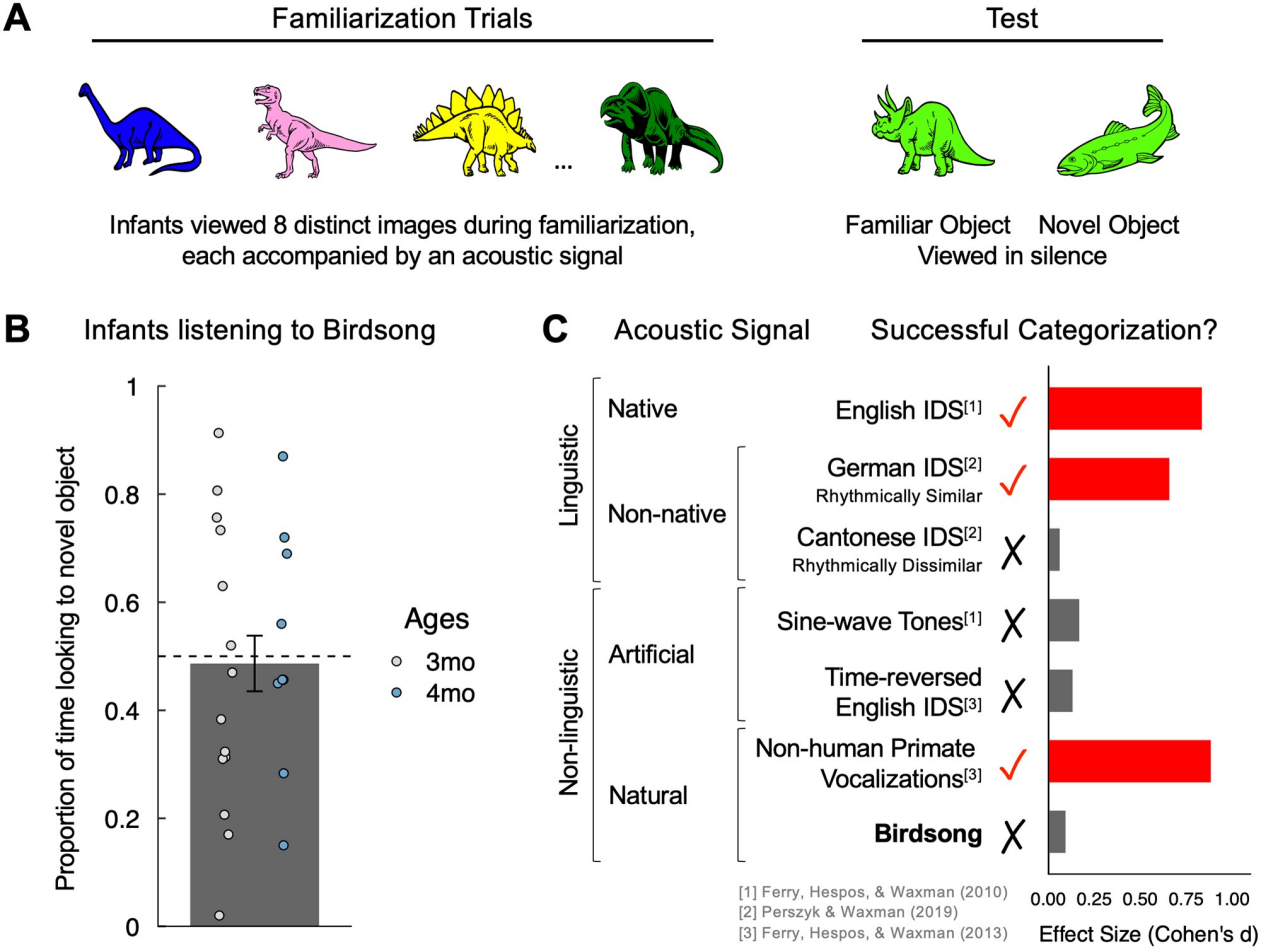
Experimental design and results from current study. (A) The experimental design of the object categorization task in the current study was identical to that of previous studies. During familiarization, each infant viewed eight distinct visual images, presented sequentially. Each image was accompanied by a pre-recorded acoustic signal. The acoustic signal accompanying the images during familiarization was the only factor that differed among studies. At test, each infant viewed two images: a new member of the now-familiar category (“Familiar Object”) and a member of a novel category (“Novel Object”). Test images were presented simultaneously, in silence. (B) Infants in the current experiment, who listened to the zebra finch song during familiarization, showed no looking preference towards either of the test objects, indicating that listening to zebra finch song does not support object categorization in 3- and 4-month-olds (*N* = 23; *p*>0.05; error bars represent ±1 SEM). (C) Object categorization outcomes for the current study (zebra finch song) and previously tested acoustic signals. Effect size for the influence of each acoustic signal on Object Categorization (mean proportion looking to the novel object compared to 50% chance) was computed separately for each age group (3- and 4-month-olds) and then averaged.

Even before infants say their first words, their ability to establish object categories is supported by language [6]. The evidence for this claim comes from a simple, yet robust object categorization task (Fig 1A) based on decades of empirical evidence [12–14]. In this task, there is an initial familiarization phase, during which infants view a series of distinct objects (e.g., images of 8 different dinosaurs), all members of a given object category (e.g., dinosaur). Immediately after the familiarization phase, infants view two new objects—one a member of the now-familiar category (e.g., another dinosaur) and the other a member of an entirely different category (e.g., a fish). If infants detect the category-based commonalities among the familiarization objects, then at test they should distinguish the novel from the familiar test image; if infants fail to detect such commonalities among the familiarization objects, then they should perform at chance levels.

This paradigm also permits researchers to examine the effect of speech and other acoustic signals on object categorization: by holding constant the objects infants view, researchers can systematically manipulate the sounds paired with the familiarization images. For infants as young as 3 or 4 months of age, listening to their native language during familiarization supports object categorization in a way that other acoustic signals do not, including other signals matched to infant-directed speech in spectral composition (e.g., time-reversed infant-directed speech) or mean pitch and duration (e.g., sine-wave tone sequences) [6, 15] (for summary, see Fig 1C). Notice that the cognitive advantage conferred by pairing visual images with the sounds of their language cannot be accounted for by appealing to associative mechanisms: in the object categorization task, all acoustic signals (infant-directed speech, time-reversed speech and tone sequences) are presented in conjunction with the very same visual images of objects, but time-reversed speech and tone sequences fail to support object categorization (for review, see [11]).

The cognitive advantage conferred by speech is itself shaped by infants’ perceptual tuning to their native language. Within the first months of life, infants have already begun to specify which human languages they will link to cognition: for 3- and 4-month-olds acquiring English as a native language, listening to German (a language that shares rhythmic and other suprasegmental properties with their native English) also supports cognition—but listening to Cantonese (a language from a different rhythmic class than English) does not [16]. This reveals that infants’ increasingly precise perceptual and neural tuning to their native language [17–20] has powerful *downstream consequences*, setting constraints that specify which language(s) infants consider as candidate links to cognition.

Crucially, however, even as infants systematically narrow the link between human language(s) and cognition, they maintain an apparently broader link for non-human vocalizations. For 3- and 4-month-old infants, listening to vocalizations of non-human primates (blue-eyed black lemur; *Eulemur macaco flavifrons*) confers the same advantage for object categorization as listening to their native language [15]. This finding is consistent with the possibility that the interface between language and cognition, which emerges surprisingly early in infants, is part of a broader template that initially encompasses not only the vocalizations of humans, but also those of non-human primates. This finding, striking in itself, also suggests that that the signals infants initially link to cognition are not driven by experience alone. By 3 to 4 months, although infants have had ample exposure to their native language, but little to no exposure to lemur vocalizations, both offer the same cognitive advantage for categorization. This suggests that as infants identify candidate links to cognition, non-linguistic vocalizations may not be subject to the same experience-based tuning parameters as linguistic signals.

Together, these findings raise a fundamental question: what are the boundary conditions on the range of non-linguistic signals that are included in infants’ initially broad template? One possibility is that infants’ initial template includes only the vocalizations of primates, our closest phylogenetic relatives, whose vocalizations may be perceptually similar enough to our own to serve as initial candidates upon which to launch the language-cognition interface. Another possibility is that infants’ initial template is sufficiently broad to include vocalizations beyond those of primates.

To address this question, we investigate the influence of birdsong (zebra finch; *Taeniopygia guttata*) on 3- and 4-month-olds’ object categorization. Our decision to focus on infants’ response to birdsong was strategic: selecting a phylogenetically distant species, whose vocal apparatus (and hence, whose vocalizations) differ from our own, offers a bold opportunity to identify a boundary on which other naturally produced non-linguistic signals, if any, support early infant cognition. If listening to birdsong supports successful object categorization, this will suggest that quite a broad range of non-linguistic vocalizations serve initially as candidate links to cognition. However, if listening to birdsong fails to support object categorization, this will suggest that the set of non-linguistic vocalizations that infants initially link to cognition may be more restricted, including only those produced by primates or perhaps mammals.

There are a number of reasons to predict that birdsong might, like human language, support infant categorization. Birdsong is the most-studied model system for human speech learning, because of behavioral, neural, and genetic similarities between the acquisition of birdsong and human speech [21–26]. Like humans and relatively few other species, songbirds are prolific vocal learners, an ability that appears to be supported by sophisticated neural circuitry in the forebrain [24, 25]. This suggests that humans and songbirds share an evolutionary history that influences how their vocalizations are produced and perceived by conspecifics. In addition, the acquisition of both birdsong and human speech employ similar molecular substrates (e.g., *FoxP2* expression [27]) and share other aspects of their ontogenesis (e.g., both birdsong and human language are socially transmitted during a sensitive period for learning [26]).

Moreover, acoustic similarities between birdsong and human speech give rise to surface-level perceptual features of birdsong that may be particularly salient to human infants. Songbirds produce complex songs that are harmonically rich and incorporate phonological syntax and prosodic properties (e.g., rhythm, pitch excursions, changes in duration, intonation-like patterns) that resemble aspects of human speech [21, 28, 29]. Converging evidence for these perceptual similarities comes from very young infants’ perceptual preferences. Infants’ preference for listening to speech, as compared to many other signals, is well-documented [30–32]. But this preference, evident in neonates, is not exclusive to speech; 3- and 4-month-olds also favor listening to vocalizations of non-human primates [30, 31] and birdsong [33]. This raises the possibility that listening to birdsong, like speech and non-human primate vocalizations, might have downstream consequences for early infant cognition, including object categorization.

At the same time, however, there are also acoustic differences between birdsong and human speech. For example, birdsong typically has a higher mean pitch than human speech and can include more than one pitch simultaneously due to the bird’s syrinx—a feature that is impossible to reproduce with the human larynx. These acoustic differences might distinguish birdsong from human speech, in a way that precludes it from the set of signals that support early infant cognition.

In sum, determining the influence of birdsong on infant object categorization will help to identify the breadth of naturally produced acoustic signals that support infant cognition.

## Methods

All procedures were approved by the Northwestern University Institutional Review Board and written informed consent was obtained from all infants’ caregivers at the beginning of each lab visit. All participants received a book and t-shirt as compensation for participation.

### Participants

Twenty-three infants were included in the final analyses (9 females; ages 2.99–4.48 months, *M* = 3.72, *SD* = 0.50). This age range is consistent with prior work with 3- and 4-month-olds using this paradigm (Ferry et al., 2010: IDS: *N* = 22, ages 3.09–4.54 months, *M* = 3.74, *SD* = 0.48; sine-wave tone sequences: *N* = 24, ages 2.57–4.63 months, *M* = 3.59, *SD* = 0.62; Ferry et al., 2013: time-reversed IDS: *N* = 24, ages 2.33–4.39 months, *M* = 3.60, *SD* = 0.66; non-human primate vocalizations: *N* = 24, ages 2.51–4.53 months, *M* = 3.70, *SD* = 0.61; Perszyk & Waxman 2019: German IDS: *N* = 31, ages 3.03–4.97 months, *M* = 4.03, *SD* = 0.61; Cantonese IDS: *N* = 38, ages 2.99–4.93 months, *M* = 4.07, *SD* = 0.60).

Another 16 participated but were excluded from analyses due to fussiness (*N* = 1) or insufficient attention during familiarization (less than 50% overall looking time; *N* = 15). This exclusion rate is consistent with prior work [6, 15].

### Materials

#### Visual stimuli

The visual stimuli are identical to those used in prior investigations of infant object categorization at 3 and 4 months [6, 15]. Line-drawn images of either dinosaurs or fish formed an eight-item familiarization set and test pair (Fig 1A). Within each familiarization set, images varied in color; within each test pair, images were matched in color. Images (∼15cm^2^) were projected onto a white screen ∼75cm from the infant’s eyes.

#### Acoustic stimuli

**Fig 2:**
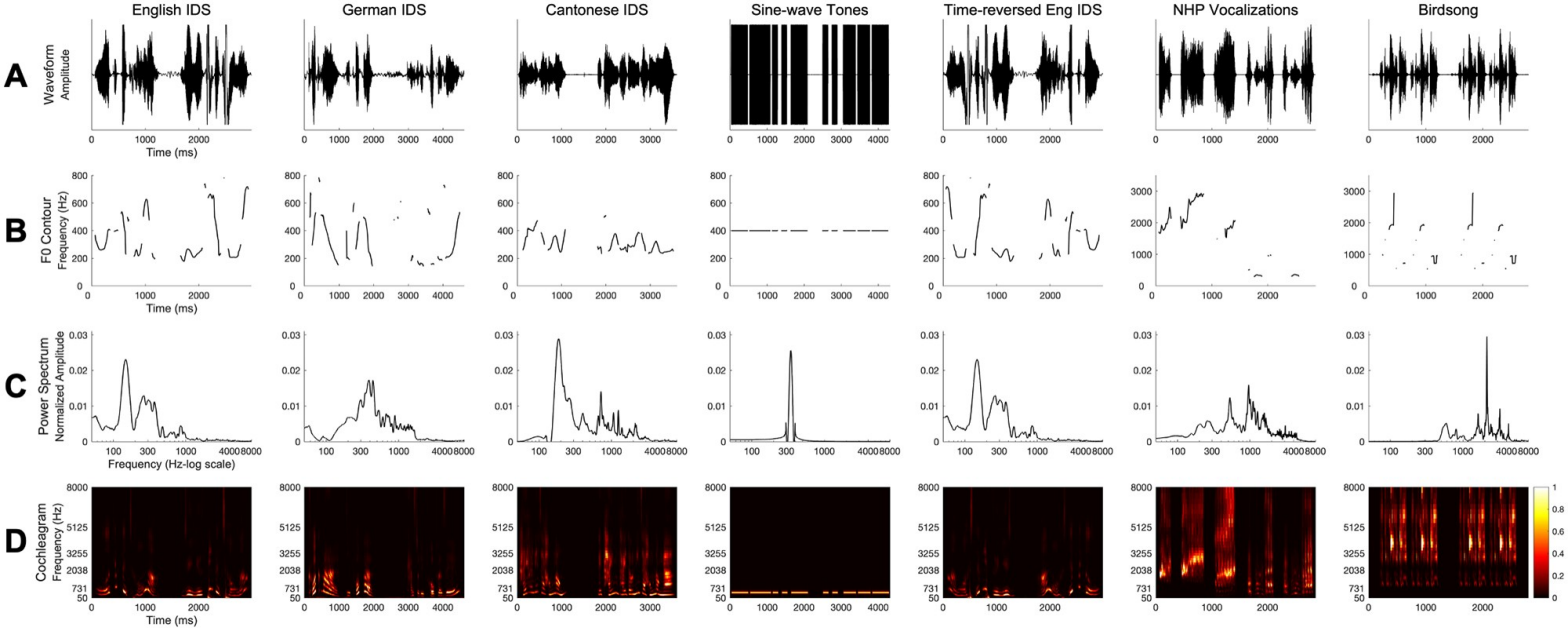
Acoustic stimuli from previous studies and current study. (A) Time-domain waveforms; (B) Fundamental frequency (F0) contours extracted using *Praat*; (C) Amplitude spectrums computed using a fast Fourier transform (FFT) algorithm; (D) Cochleagrams computed using *ERBFilterBank*.*m* [36] and *gammatoneFast*.*m* [37] to construct the weighting matrix to convert time-frequency spectrograms into 64 gammatone-filter approximations to match the human cochlea’s frequency sub-bands.

Only the acoustic stimuli differed from those used in prior investigations. Here, the acoustic signal presented in conjunction with each familiarization image was a recording of a mating song produced by a male zebra finch (*Taeniopygia guttata*). We selected this species of bird because zebra finches produce complex songs that are strongly harmonic, incorporating many acoustic features that result in perceptual attributes salient to humans (e.g., pitch, loudness, and tempo). Moreover, male zebra finches have been instrumental in investigations of avian vocal learning and its neurobiological bases (for reviews see, [22, 34]). The zebra finch stimulus created for the current investigation was comprised of a recorded song, played twice, to achieve a duration (2.8s in total) to approximate the duration of acoustic stimuli in previous studies [6, 15]. A comparison of the durations and pitch of the acoustic stimuli in prior studies and the current study can be found in Table 1 and Fig 2. The song was played from a hidden speaker, located 56cm below the center of the screen.

**Table 1:** Duration and pitch of acoustic stimuli from previous studies and current study.

	Duration	Pitch Mean (SD)	Pitch Range
**Linguistic**			
**English IDS** [6] Native Language	2979ms	360.163(168.7) Hz	177.932–782.064Hz
**German IDS** [16] Rhythmically Similar Non-native Language	4608ms	337.085 (164.1) Hz	142.339–785.674Hz
**Cantonese IDS** [16] Rhythmically Dissimilar Non-native Language	3595ms	313.091 (65.1) Hz	231.291–509.552Hz
**Non-linguistic**			
**Sine-wave Tones** [6]	4310ms	400.000 (0) Hz	400.000Hz
**Time-Reversed English IDS** [15]	2979ms	349.720 (166.7) Hz	128.124–742.231Hz
**NHP Vocalizations** [15]	2851ms	1623.107 (939.3) Hz	140.761–2948.167Hz
**Birdsong** (Current study)	2799ms	1326.409 (604.9) Hz	536.509–2949.823Hz

Acoustic stimuli from previous studies investigating the influence of linguistic (English, German, and Cantonese infant-directed speech (IDS) [6,16]) and non-linguistic acoustic signals (sine-wave tone sequences [6], time-reversed speech and non-human primate (NHP) vocalizations [15], and zebra finch song (Birdsong; current study) on infant object categorization. All values were extracted using *Praat* [35].

### Procedure

As in all prior studies using this paradigm, infants were seated on a caregiver’s lap facing the screen. Caregivers, who wore opaque glasses, were instructed not to talk to their infants or influence their infant’s attention in any way. Infants’ behavior was recorded by a video camera hidden below the screen.

The experimental design was identical to that used in previous studies investigating the range of acoustic signals that promote object categorization in 3- and 4-month-old infants [6, 15, 16], with only a single exception: the acoustic stimulus accompanying each familiarization image was the vocalization of the zebra finch. During the familiarization phase, infants viewed eight images from a single category (e.g., dinosaurs), presented one at a time, in random order on a screen. At test, infants viewed two new images, presented in silence–a new member of the familiar category (e.g., another dinosaur) and an object from a novel category (e.g., a fish). If listening to the zebra finch song supports infants’ object categorization, then they should detect the category-based commonalities among the objects presented during the familiarization phase (e.g., dinosaurs), and should therefore distinguish the familiar from novel test objects, expressing this behaviorally with a reliable visual preference at test (for review see [12]).

#### Familiarization phase

Visual stimuli (either 8 distinct dinosaurs or 8 distinct fish, counterbalanced across participants) were presented on alternating sides of the screen (20s each). The left/right position of the first familiarization image was counterbalanced across infants. As in all previous implementations of this task, the acoustic stimulus was presented as each image appeared and was repeated after 8s.

#### Test phase

All infants, regardless of familiarization category, viewed the same two images at test. The two images appeared side-by-side, separated by 11cm, in silence, and remained visible until infants accumulated 10s of looking at the test images, as coded by an online coder. The left/right position of the familiar and novel test images was counterbalanced across infants.

### Coding

Infants’ left-right eye gaze directions were coded offline using frame-by-frame software [38] by trained coders blind to acoustic condition. A second observer re-coded 20% of the videos. Reliability between observers was high (*r* = 0.90, *p* < 0.01).

### Data analysis

All included infants, regardless of familiarization category, were combined for all analyses.

To derive a measure of visual preference we calculated, for each infant, the proportion of looking time devoted to the novel object (total accumulated looking to the novel test object, divided by total accumulated looking to both test objects) during the first 10s of accumulated looking (as coded offline) to test images.

## Results

Infants were readily engaged in this task. During the familiarization phase, infants’ visual attention to each object was comparable to that of their age-mates in prior investigations. There was no statistical difference in infants’ looking time to each familiarization object, whether objects were presented in conjunction with the zebra finch vocalization or with infant-directed speech (from [6]; independent t-tests were all *p* > 0.05). This is consistent with prior evidence that infants’ visual attention during familiarization is comparable, whether visual stimuli were accompanied by linguistic or non-linguistic stimuli [6, 15]. This provides assurances that differences in infants’ categorization at test are not likely attributable to their visual attention during familiarization.

At test, infants revealed no preference for either test object (novelty preference: *M* = 0.487, *SD* = 0.247); their performance did not differ from chance (one-sample t-test: *t*(22) = 0.259, *p* = 0.798; BF_10_ = 0.225; Fig 1B). This suggests that listening to the zebra finch song failed to promote 3- and 4-month-olds’ object categorization.

Moreover, there was no influence of age on infants’ categorization behavior. Prior evidence has revealed an intriguing developmental pattern for signals that support infant categorization (i.e., their native language and non-human primate vocalizations): younger infants demonstrate a looking preference towards the familiar object, while older infants demonstrate a looking preference towards the novel object [6, 15]. This familiarity-to-novelty shift is a well-documented developmental feature of looking-time paradigms [12, 13]. For birdsong, we did not observe this developmental shift, as there was no relationship between age and visual preference at test (Pearson correlation: *r*(21) = 0.088, *p* > 0.05). Instead, the results mirror those of other signals that fail to support infant categorization at these ages (i.e., sine-wave tone sequences and time-reversed speech). This finding lends further support to the interpretation that birdsong does not support early infant categorization.

## Discussion

Infants failed to form object categories in the context of listening to zebra finch song. This outcome, which contrasts with infants’ success while listening to non-human primate vocalizations [15], sharpens the boundary conditions on the range of non-linguistic signals that initially engage infants’ core cognitive process of categorization. It provides the first evidence that not all naturally produced non-linguistic vocalizations support infants’ object categorization.

This new finding also sheds light on the role of exposure in identifying candidate links to infant object categorization. For linguistic signals, exposure is clearly instrumental: it not only guides infants as they tune perceptually to the sounds of their native language(s) [17–20], but also has downstream consequences for which languages infants continue to accept as candidates to link to categorization. By 4 months, infants have begun to narrow the set of human language(s) that support categorization [16]. But for non-linguistic signals, exposure appears less crucial. Although infants have had little exposure, if any, to either non-human primate vocalizations or birdsong, they accept vocalizations of the former, but not the latter, as candidates to link to cognition. This suggests that exposure-based tuning may be less critical in the selection of non-linguistic sounds than the selection of linguistic sounds that 3- and 4-month-olds link to categorization.

The data reported here also raise compelling questions about the range of acoustic signals that support infants’ earliest cognitive capacities. For example, it remains unknown which acoustic features, singly or in combination, infants rely upon in identifying which sounds they will link to cognition. The current results provide some insight. For example, duration is unlikely to prove diagnostic: although the acoustic stimuli tested thus far have been comparable in duration, these stimuli exert different effects on infants’ object categorization. It also seems unlikely that pitch can account for the observed pattern of effects: the mean fundamental frequency (f0; perceived as pitch) of the birdsong recording was comparable to that of the non-human primate vocalization—yet these stimuli exerted different cognitive consequences. Although the lower range of the non-human primate vocalization overlapped more with the infant-directed speech than did birdsong, the range of the birdsong f0 fell between the f0 range of the stimuli that promote object categorization in infants (native and rhythmically similar non-native infant-directed speech, non-human primate vocalization). Therefore, it remains possible that the non-linguistic vocalizations that initially support infant categorization have a pitch range more similar to human speech. Additional behavioral evidence—from infants’ categorization in the context of vocalizations of additional species, as well as additional samples of vocalizations from these tested species—may reveal which acoustic features contribute to the selection of signals that initially support infant categorization.

Perhaps most importantly, future work is also needed to further investigate whether infants’ earliest link to cognition is sufficiently broad to include the vocalizations beyond those of primates (e.g., non-primate mammals), or whether only the vocalizations of primates are included in this privileged set. Identifying the cognitive consequences of listening to vocalizations from species ranging systematically in phylogenetic distance from humans will offer insight into the boundary conditions on the signals that serve as initial, candidate links to cognition. If these boundaries mirror evolutionary distance, then it will be important to consider whether and how infants’ responses can be accounted for by similarities or differences in physiology that yield vocalizations that range in perceptual similarity to our own. Addressing these matters will advance our understanding of the ontogenetic and phylogenetic antecedents to human language acquisition and its quintessentially human link to cognition.
